# Between-hand difference in ipsilateral deactivation is associated with hand lateralization: fMRI mapping of 284 volunteers balanced for handedness

**DOI:** 10.3389/fnhum.2015.00005

**Published:** 2015-02-06

**Authors:** N. Tzourio-Mazoyer, L. Petit, L. Zago, F. Crivello, N. Vinuesa, M. Joliot, G. Jobard, E. Mellet, B. Mazoyer

**Affiliations:** GIN UMR5296, CNRS CEA Université de BordeauxBordeaux, France

**Keywords:** hemispheric specialization, lateralization, handedness, left-handers, finger tapping fMRI, primary motor area, dominance, inhibition

## Abstract

In right-handers (RH), an increase in the pace of dominant hand movement results in increased ipsilateral deactivation of the primary motor cortex (M1). By contrast, an increase in non-dominant hand movement frequency is associated with reduced ipsilateral deactivation. This pattern suggests that inhibitory processes support right hand dominance in right-handers and raises the issues of whether this phenomenon also supports left hand preference in left-handers (LH), and/or whether it relates to asymmetry of manual ability in either group. Thanks to the BIL&GIN, a database dedicated to the investigation of hemispheric specialization (HS), we studied the variation in M1 activity during right and left finger tapping tasks (FTT) in a sample of 284 healthy participants balanced for handedness. An M1 fMRI localizer was defined for each participant as an 8 mm diameter sphere centered on the motor activation peak. RH exhibited significantly larger deactivation of the ipsilateral M1 when moving their dominant hand than their non-dominant hand. In contrast, LH exhibited comparable ipsilateral M1 deactivation during either hand movement, reflecting a bilateral cortical specialization. This pattern is likely related to left-handers’ good performances with their right hand and consequent lower asymmetry in manual ability compared with RH. Finally, inter-individual analyses over the whole sample demonstrated that the larger the difference in manual skill across hands, the larger the difference in ipsilateral deactivation. Overall, we propose that difference in ipsilateral deactivation is a marker of difference in manual ability asymmetry reflecting differences in the strength of transcallosal inhibition when a given hand is moving.

## Introduction

An important hypothesis regarding deactivation of the ipsilateral primary motor cortex (M1) during hand movement has been proposed by Hayashi et al. (2008). Investigating healthy right-handers (RH) with functional magnetic resonance imaging (fMRI) these authors have shown that the activity of the ipsilateral M1 during unimanual movement varies with the hand used (Hayashi et al., 2008). They demonstrated that, at low movement frequency, the contralateral M1 was moderately activated and the ipsilateral M1 was deactivated, regardless of the hand used. By contrast, increasing movement frequency was associated with a stronger ipsilateral deactivation during right-hand movement. Conversely, during left hand movement, the dominant left M1 participated in left hand motor control and became less deactivated, as this activity counters the IHI from the right M1 (Hayashi et al., 2008). In RH there have been other fMRI reports of ipsilateral deactivation during dominant (right) finger (Nirkko et al., 2001) or arm (Vidal et al., 2014) movements but not during similar movements of the non-dominant limb. According to Hayashi, this opposite pattern of deactivation in motor cortices controlling the dominant and non-dominant hands “demonstrate the dominance of the left M1 in both ipsilateral innervation and transcallosal inhibition in right-handed individuals” (Hayashi et al., 2008). Actually such assertion is based not only on functional imaging observations of ipsilateral deactivation during hand or arm movement, but also on observations with paired-pulse transcranial magnetic stimulation (TMS) of decrease in excitability of ipsilateral motor potential after stimulation of the contralateral M1, named the ipsilateral silent period. This inter-hemispheric inhibition (IHI) from contralateral M1 onto ipsilateral M1 is involved in the control of unilateral movements (Tazoe and Perez, 2013). The anatomical support for this phenomenon is the callosal connections joining homotopic M1 areas.

However, in order to prove that the strength of ipsilateral deactivation is related to manual dominance, one must investigate whether a similar phenomenon can be observed in left-handers (LH). LH, although less lateralized in terms of manual ability and manual preference (MP) than RH (Peters and Durding, 1978; Doyen et al., 2001; Mellet et al., 2014), do exhibit MP (i.e., the behavioral expression of motor hemispheric specialization (HS)). The observation of a mirrored pattern in LH would provide strong support to the hypothesis that the larger ipsilateral deactivation of the M1 hand area when the dominant hand is moving is a marker of IHI supporting hand motor dominance.

Findings regarding such a decrease in activity in the ipsilateral M1 during hand movements are missing in LH. Apart from Hayashi’s (2008) study, previous functional imaging investigations in LH have looked only at activated voxels in small samples of participants (Kawashima et al., 1997; Verstynen et al., 2005; Klöppel et al., 2007; Grabowska et al., 2012). One paired-pulse TMS study of IHI indicates that common mechanisms may support both right and left hand preference. Bäumer et al. (2007) showed larger motor evoked potential reduction (corresponding to larger IHI) when the conditioning TMS pulse was applied over the M1 of the dominant hemisphere, in both LH and RH. Note that participants in this TMS study (20 per group) were selected as having a strong MP according to the Edinburgh Score (ES; Bäumer et al., 2007). However, their observation is challenged by another TMS report showing that, while increased inhibitory processing is observed in RH after the application of a TMS pulse to the left hemisphere, the mirrored situation is not observed in LH, which suggests a different organization for motor dominance (Reid and Serrien, 2012). However, this last study was conducted in groups of only 8 participants, and although participants were selected as strongly lateralized, these results must be considered with caution because LH express a larger variability in their strength of MP.

Very recently, dynamic causal modeling (DCM) was applied to investigate whether effective connectivity during unimanual movements differs with MP (Pool et al., 2014). In this work, comparison of activation between RH and LH during unimanual tasks failed to demonstrate any difference in contralateral M1 activity for either hand and showed that variation of M1 activity with movement frequency was independent of handedness, as already reported during simple motor tasks (Solodkin et al., 2001). DCM analyses revealed stronger inter-hemispheric connectivity between supplementary motor areas (SMAs) and a stronger inhibitory influence of the ipsilateral SMA on the ipsilateral M1 during movement of the dominant hand than of the non-dominant hand in RH; this phenomenon was also stronger in RH than in LH (Pool et al., 2014). These results highlight that inhibitory modulation of ipsilateral motor cortices (rather than contralateral activations) is a likely support of manual dominance.

As a whole, these results obtained in relatively limited samples of participants do not allow definitive conclusions regarding the commonalities and/or differences in M1 variations during unimanual movement in LH and RH. To investigate these questions further, we took advantage of the BIL&GIN database^1^, which is dedicated to the investigation of HS. The BIL&GIN contains records from a sample of 453 adult participants enriched in LH (45%, *N* = 205) as compared to the general population. For each subject, socio-demographic data, hand and eye laterality, family handedness, and cognitive abilities in the language, motor, visuo-spatial, and numerical domains have been recorded. T1-MRI and diffusion tensor imaging (DTI) data were also acquired, as well as resting-state functional MRI. Task-evoked functional MRI was performed in a sub-sample of 303 subjects (157 LH) using a customized functional battery of 16 cognitive tasks exploring the same three cognitive domains. Here, we studied in 284 individuals of this sub-sample the variation of activity in the right and left hand M1 during right and left self-paced finger-tapping tasks (FTT) with functional localizers detected in each individual. Participants were trained to a 2-Hz tapping frequency because this frequency is optimal for the detection of both contralateral activation and ipsilateral deactivation of the M1 hand area (Hayashi et al., 2008). As a first step, we compared the patterns of M1 activity in RH and LH, testing whether mirrored patterns of activation and/or deactivation were associated with differences of MP. As a second step, in order to establish whether these regional patterns of activity were related to HS for hand motor control, we searched for an association between MP and the activity in the ipsilateral M1 region during either hand movement, and the existence of a relationship between ipsilateral deactivation during either hand movement and participants’ asymmetry regarding manual skill.

## Materials and methods

### Participants

In accordance to French regulation, a local ethical committee approved the study; the volunteers provided informed consent and received an allowance for their participation. From the 303 participants of the BIL&GIN, 288 completed both tasks adequately and were free of movement artifacts in both tasks. Manual laterality definition was based on self-reported handedness, i.e., by asking the participants whether they considered themselves right-handed, left-handed or forced right-handed. The 15 excluded participants were 4 RH and 11 LH. We also excluded the 4 forced RH participants because of potential variation in hand motor dominance mechanisms in this case. The study sample was thus of 284 participants (age: 25 ± 6 years, 140 women).

Among the 284 participants, 142 considered themselves RH (71 men), and 142 considered themselves LH (73 men). RH and LH had significantly different mean ages (RH: 26.8 ± 5.9 years, LH: 24.4 ± 6.0 years, *p* = 0.0006, *t*-test) and levels of education (RH: 16.1 ± 2.2 years, LH: 15.1 ± 2.3 years, *p* = 0.0002, *t*-test). Due to the balanced study design, there was no significant association between self-reported handedness and sex in the study sample.

Strength of MP was evaluated using 9 of the 10 items of the Edinburgh inventory (EI; Oldfield, 1971). The “broom” item was discarded because this tool is no longer familiar to young people.

We used a finger-tapping test (FTT) to assess manual skill (Peters and Durding, 1978). Each participant, while keeping his or her wrist on the table, had to hit a button with his or her index finger as many times as possible during 10 s. For each index finger, measures were repeated three times and then averaged. Manual skill asymmetry (MSA) was calculated as the right minus left tap numbers (Tap), from which an MSA index was derived through normalization for the total number of taps as:

MSA index = 100* (RTap−LTap)/(RTap+LTap),

where RTap (respectively, LTap) represents the average right (respectively, left) index finger tapping score. Compared to the MSA, the MSA index takes into account potential differences in the total number of taps, what can be important when comparing groups that differ on that total number of taps.

### MRI data acquisition and analysis

#### Anatomical MRI

MRI was performed on the same Philips Achieva 3 Tesla MR scanner for all participants. The acquisition protocol, which lasted 30 min, included a high-resolution 3D T1-weighted sequence (3D-fast field echo(FFE)-turbo field echo(TFE); TR = 20 ms; TE = 4.6 ms; flip angle = 10°; inversion time = 800 ms; TFE = 65; sense factor = 2; matrix size = 256 × 256 × 180 mm^3^; 1 mm^3^ isotropic voxel size) and a T2*-weighted multi-slice FFE (TR = 3,500 ms; TE = 35 ms; flip angle = 90°; sense factor = 2; 70 axial slices; 2 mm^3^ isotropic voxel size).

Each participant’s T1-weighted volume was spatially normalized using a specific cerebral tissue template built from the T1-weighted images of 80 participants (including 40 men). The template had been acquired using the same scanner and acquisition parameters (Template resolution of 1 × 1 × 1 mm^3^ voxels; bounding box, *x* = −90 to 90 mm, *y* = −126 to 91 mm, *z* = −72 to 109 mm), normalized into the Montreal Neurological Institute (MNI) stereotaxic space (Ashburner and Friston, 2000). T1-weighted volumes were processed using the statistical parametric mapping version 5 (SPM5) “segment” procedure with default parameters allowing segmentation of gray matter, white matter, and cerebrospinal fluid components for each participant. The Total Intracranial Volume (TIV) was computed as the sum of the three component tissue volumes.

#### Functional MRI

##### Finger tapping tasks.

Finger tapping was also used to assess the asymmetry of the hand motor system. The participant held a fiber optic response pad in each hand (Current Designs Inc, Philadelphia, PA, USA). Depending on the orientation of a symbolic cue presented at the center of the screen (arrowhead “>” or “<”), the participant had to tap his right or left index finger on the response pad at 2.0 Hz as regularly as possible. Participants were instructed to perform this rhythmic FTT for as long as the visual cue (> or >) was displayed (i.e., 12 s). The FTT was alternated with a reference task where the participants had to fixate a central crosshair. Both arrowheads and crosshair covered the same 0.8° × 0.8° visual angle. Motor responses from all but one participant were collected from either hand using the two fiber optic response pads. Before scanning, participants were trained to perform the FTT with the help of a metronome set at a frequency of 2 Hz.

The fMRI paradigm randomly alternated six 12-s blocks of finger tapping (3 right and 3 left) with six 12-s blocks of central fixation crosshair reference task within a run that also included 4 blocks of 16-s visually guided saccadic eye movements (VGS) along with 4 blocks of 16-s reference central fixation task. Functional images were acquired with a T2*-weighted echo-planar sequence (T2*-echoplanar imaging (EPI); 72 volumes; TR = 2 s; TE = 35 ms; flip angle = 80°; 31 axial slices; 3.75 mm^3^ isotropic voxel size) covering the same field of view as the T2*-FFE acquisition.

##### Whole brain analysis of the activation patterns of left and right finger tapping tasks.

This analysis was completed with an in-house pipeline including SPM5 routines.^2^ First, T2*-EPI images were corrected for slice timing differences and motion (6 parameters: 3 translations and 3 rotations), registered to the T2*-FFE volume, and then spatially normalized combining the T2*-FFE with the T1-weighted registration matrix and the T1-weighted stereotaxic normalization matrix. Normalized T2*-EPI volumes were then spatially smoothed using a 6-mm full width at half-maximum Gaussian kernel. Finally, using white matter and cerebrospinal fluid fMRI time series (average time series of voxels belonging to each tissue class), the six motion parameters and the temporal linear trend were regressed out of the fMRI data.

Next, three regressors were included in a general linear model. The right finger tapping task (RFTT) regressor included the 3 blocks of right hand finger tapping and their 3 reference blocks, the left finger tapping task (LFTT) regressor included the 3 blocks of the left hand finger tapping and their 3 reference blocks, the saccadic eye movement task regressor included the 4 blocks of VGS and their 4 reference blocks. Each regressor was constructed with the blocks of interest modeled by boxcar functions corresponding to paradigm timing and convolved with a standard hemodynamic response function. The multiple regression method allows obtaining estimates of activity levels for each task (RFTT, LFTT, VGS).

A second-level analysis was computed to uncover activations triggered by RFTT and LFTT tasks and those shared in common applying a conjunction analysis, and to compare the task-related activation patterns of RH to those of LH (SPM5, and voxel-level statistical thresholds was set at *p* < 0.05, family-wise error-corrected).

##### Localizers of hand motor area (M1_ROI).

For each participant, using an inhouse software, we detected the coordinates of the peak of the cluster that had the largest significant activation in the sensorimotor hand area contralateral to the moving hand. In order to perform automatic detection of these peaks, the search was started at the coordinates of the peak obtained on the mean activation map including all participants, in each hemisphere contralateral to the movement. Then, for each participant, left and right M1_ROIs were defined as the intersection between 8 mm diameter spheres centered on the peaks detected in the sensorimotor hand area and the activation mask during contralateral movement (threshold *p* < 0.05, uncorrected). Blood oxygen level-dependent (BOLD) mean variations within each M1_ROI were computed for each task-related contrast map and each hemisphere. As a control, we calculated the percentage of the 8 mm diameter sphere that intersected with activated area in individual contrast set at 0.05 (uncorrected threshold), the mean sphere overlap with activation was 99.96 ± 0.35% in the right M1_ROI, range [97–100], during LFTT and 99.87± 1.60% [76–100] in the left M1_ROI during RFTT.

### Statistical analysis

All statistical procedures on ROIs were conducted using the JMP11 Pro software package.^3^

#### Behavioral control

Using repeated-measures analysis of variance (ANOVA), we checked that the actual tapping frequency during fMRI acquisition did not differ between tasks. We also searched for possible effects of age, educational level, and sex, as well as for respective interactions between the side of finger movement and sex or MP.

#### Manual preference and asymmetry in manual ability

Because RH and LH differ regarding manual ability lateralization (Peters and Durding, 1978; Doyen et al., 2001) and strength of MP (Corey et al., 2001; Mellet et al., 2014), self-reported RH and LH in the present study were characterized for these manual lateralization items. We conducted repeated-measures ANOVA of the RTap and LTap number to search for effects of MP, sex, and Side of finger tapping and their Side × MP and sex × MP interactions. Sex, age and educational level were entered into the model as covariates. To complete the *post hoc* analysis regarding side effect or interaction with side, we applied an ANOVA to the difference of tap (MSA) and to the MSA index that allows taking into account differences in the total number of taps, that can differ, in particular, with sex, men producing a larger number of taps than women.

#### Peak localization

Absolute values of *x*, *y*, and *z* average coordinates of the right- and left-hemisphere locations of the M1_ROI local maxima were compared in the entire sample using paired *t*-tests. Average peak coordinates were also compared between RH and LH and included TIV in the model to take into account a possible size of the brain effect.

#### BOLD activity during RFTT and LFTT in M1_ROI

Repeated-measures ANOVAs were used to investigate the effects of MP, side of finger movement, and their interaction on BOLD variations in M1_ROIs during the RFTT and LFTT tasks. Separate ANOVAs were conducted for the respective M1_ROIs contralateral to and ipsilateral to the side of movement. In a second step, we investigated whether the amplitude of deactivation was associated with asymmetry in manual ability as assessed with the MSA index. We conducted a repeated-measures ANOVA on BOLD values within M1_ROIs ipsilateral to movement, searching for an interaction between side of movement and MSA index. Sex, age, and educational level were included as covariates.

## Results

### Manual preference and ability

Sample average EI scores were 93.6 ± 11.3 and −65.6 ± 38.5 for RH and LH, respectively as defined by their self-report. These scores reflect the well-known stronger lateralization of RH individuals and the typically larger variability in MP strength of LH individuals. There was no effect of sex on EI scores in either handedness group (RH: *p* = 0.27, LH: *p* = 0.97). Note that there was a significant difference regarding variance in MP strength between LH and RH; LH had a much larger variance (Welch’s test, SD RH: 11.3, LH: 38.3; *p* < 0.0001).

**Figure 1:**
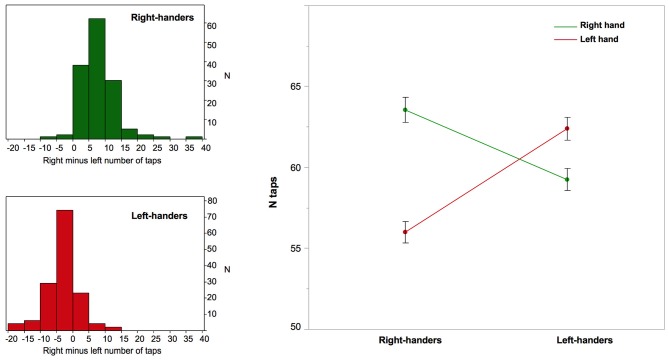
**Right-hand and left-hand manual ability and right minus left asymmetry in finger taps corresponding to Manual Skill Asymmetry (MSA) in right- and left-handers**. Left column: the distribution of the MSA in right-handers and left-handers; Right column: the graph that illustrates the significant interaction between the side of finger tapping (right in green, left in red) and manual preference regarding the number of finger taps. Note that while right-handers exhibit a highly significant difference in number of taps because of a 12% decreased performance of the non-dominant hand there is no such a large difference in left-handers, who exhibit only a 5% decrease in non-dominant hand performance. Note that the between-hand difference was significant in both groups (*p* < 0.0001), but was significantly greater among right-handers (*p* = 0.0001). Error bars represent one standard error from the mean.

The MSA index sample average and standard deviation were 6.28 ± 4.30 for RH and −2.64 ± 3.94 for LH. MSA distribution did not strictly follow a normal distribution in either handedness group but deviation from normality was due to some degree of leptokurtosis rather than to some skewness (Figure 1). As shown by others, (M)ANCOVA is robust to such modest normality violation, especially for large sample size (Olejnik and Algina, 1984). The repeated-measures ANOVA of RTap and LTap revealed a significant side × MP interaction (*p* < 0.0001) caused by a larger RTap minus LTap difference in RH than in LH. *Post hoc* analysis on MSA confirmed that this larger difference in RH was caused by the poor performances of RH during left hand tapping (RTap: RH: 63.5 ± 9.1, LH: 59.2 ± 8.2; LTap: RH: 56.0 ± 7.8, LH: 62.4 ± 8.4; *p* < 0.0001; Figure 1). The same repeated-measures ANOVA revealed a significant effect of sex (*p* < 0.0001), a significant side × sex interaction (*p* < 10^−4^), and a trend toward a side × sex × MP interaction (*p* = 0.09). This triple interaction was significant when the MSA index (rather than the MSA) was analyzed, accounting for a larger number of taps in men, (*p* = 0.01); it also indicates that both RH and LH women were, on average, more strongly lateralized than men (MSA index: RH women: 6.82 ± 3.77, LH women: −3.37 ± 3.23, RH men: 5.72 ± 4.71, LH men: −1.94 ± 4.43).

### fMRI task execution control

Movement side, MP, and their interaction had no significant effects on the mean frequencies of finger tapping recorded during fMRI acquisition (RH: RFTT = 2.17 ± 0.38 Hz, LFTT = 2.17 ± 0.39 Hz, LH: RFTT = 2.24 ± 0.43 Hz, LFTT = 2.26 ± 0.41 Hz). There was a significant effect of sex without any interaction with task or side: men had a slightly larger frequency than women (Men mean FTT = 2.27 ± 0.42 Hz, women mean FTT = 2.15 ± 0.36 Hz; *p* = 0.007). Note that there was no effect of age or educational level.

### Whole brain analysis of LFTT and RFTT activation patterns

#### Whole sample analysis

**Figure 2:**
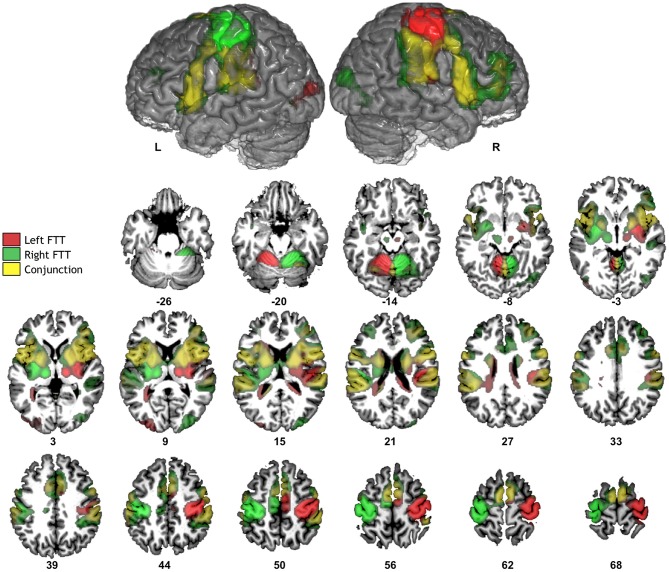
**Significant fMRI average activation pattern during left FTT (red), right FTT (green), and their conjunction analysis (yellow) among the 284 participants**. The top row provides the left and right hemisphere projections of activation superimposed onto a typical individual of the BIL&GIN anatomical template. The bottom rows depict corresponding axial slices (*p* < 0.05, family-wise error-corrected).

Contrast maps between each hand movement and fixation clearly revealed activations contralateral to movement (Figure 2, Table 1). These specific activations were present in the sensorimotor cortices and peaked at the level of the rolandic gyrus (which hosts the hand motor representation); in the most posterior part of the SMA, corresponding to the SMA proper; in the most posterior part of the insula (which hosts the supplementary sensory cortex); in the inferior and posterior parts of the lenticular nuclei; and in the posterior thalamus. There was also activation in the ipsilateral cerebellum.

**Table 1:** **Whole brain activation maps during right and left finger tapping tasks (FTT)**.

Anatomical location	*x*	*y*	*z*	*T*-value	*x*	*y*	*z*	*T*-value
Right FTT
Precentral gyrus	−40	−20	54	53.3
Post. Suppl. motor area	−6	−14	58	13.9
Ant. Suppl. motor area	−6	−4	56	27.6	2	2	64	28.4
Inferior frontal gyrus					48	10	4	29.0
Post. insula	−38	2	2	25.4	40	6	2	26.6
Rolandic operculum	−44	−28	20	29.0
Supramarginal gyrus					56	−32	50	21.3
Middle occipital gyrus					32	−94	14	12.7
Putamen	−26	−2	6	29.0	22	2	8	20.5
Thalamus	−16	−20	4	24.9
Cerebellar hemisphere	−24	−60	−18	12.7	14	−50	−18	49.9
Left FTT
Precentral gyrus					38	−20	52	55.7
Post. Suppl. motor area					8	−18	50	13.3
Ant. Suppl. motor area	−2	−2	64	22.3	4	0	64	24.5
Middle cingulate gyrus					6	10	42	11.8
Inferior frontal gyrus	−50	8	4	21.1	48	10	4	26.3
Insula	−38	4	2	21.0	40	0	14	29.0
Rolandic operculum					46	−22	20	28.6
Postcentral gyrus	−56	−20	20	14.9
Supramarginal gyrus	−60	−22	46	14.7
Post. sup temporal gyrus	−48	−38	20	18.3
Middle occipital gyrus	−32	−96	12	11.8
Putamen	−24	2	6	17.3	24	0	10	23.8
Thalamus					16	−18	6	23.1
Cerebellar hemisphere	−16	−52	−20	52.5	26	−58	−20	14.0

*Threshold set to p < 0.05 corrected for family-wise error (L: left; R: right; ant.: anterior; post.: posterior, Suppl. : supplementary)*.

Conjunction analysis highlighted significant bilateral activations in the basal ganglia, known to be involved in motor sequence learning (Doyon et al., 2009); in the rolandic operculum extending internally towards the mid and anterior parts of the insula and posteriorly to the *planum temporale*; in the *pars opercularis* of the inferior frontal gyri; and in the anterior part of the SMA, corresponding to the pre-SMA. Both tasks also involved the cerebellar vermis and the right middle frontal gyrus. There was little involvement of lateral premotor regions, which was not unexpected because of the self-paced nature of the tasks used in this study, as opposed to other studies in which hand movements were externally paced (Pool et al., 2014).

#### Comparison between RH and LH

**Figure 3:**
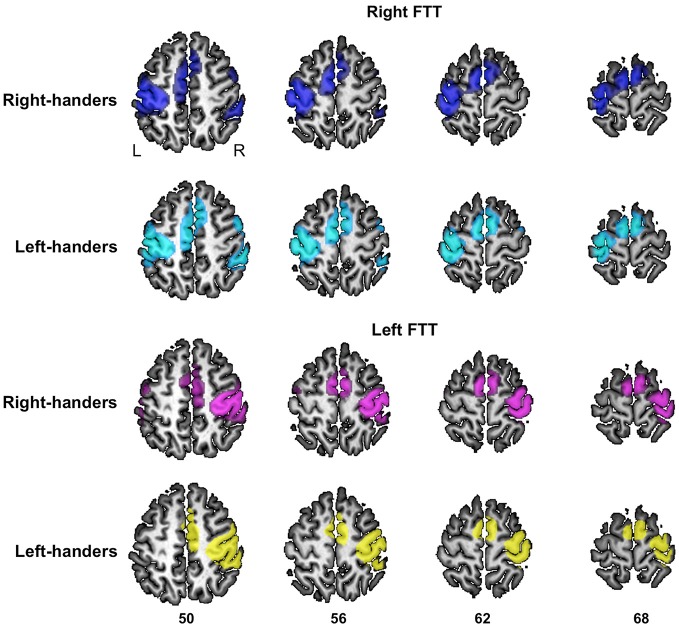
**Significant fMRI average activation pattern during left FTT and right FTT presented separately in right-handers and left-handers**. The left and right hemisphere activation are superimposed onto a typical individual of the BIL&GIN anatomical template. The *z* coordinate of the axial slices is provided (*p* < 0.05, family-wise error-corrected).

During right-hand movement, there was no difference between the contrast maps of RH and LH. During left-hand movement, RH exhibited larger left hemisphere activations in the inferior part of the postcentral sulcus (*x* = −56, *y* = −22, *z* = 42, cluster extent = 28 voxels, *t* = 5.32) and at the junction between the superior frontal and the precentral sulci corresponding to the location of the frontal eye fields (FFE) (*x* = −26, *y* = −12, *z* = 54, cluster extent = 22 voxels, *t* = 5.31) (Beauchamp et al., 2001). By contrast, there were no areas in which LH exhibited greater activation than RH during left-hand movement. As an illustration we also provide the activation maps of left and RH separately at the level of the rolandic genu at the same threshold (*p* < 0.05, FWE voxel-level corrected, Figure 3).

### Analysis of M1_ROI hand area activation

#### Locations of M1 hand area activation maxima

**Figure 4:**
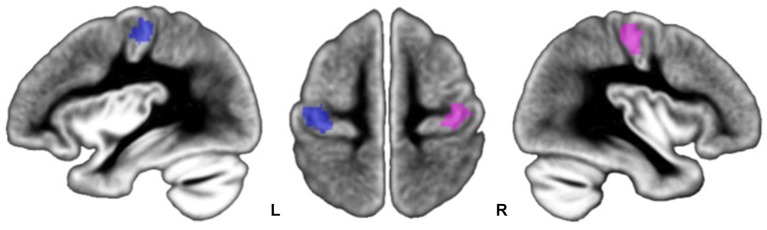
**Location of participants’ peaks during left FTT (pink dots) and right FTT (blue dots) superimposed onto the BIL&GIN template**. Note that peak locations are centered onto the *genu* of the rolandic sulcus, an anatomical marker of the location of the M1 hand area.

Mean and SD of stereotaxic coordinates of individual peaks of activation for the M1_ROIs are provided in Table 2 and illustrated in Figure 4. There was a small but significant difference in M1_ROI peak location between the two hemispheres (approximately 1 mm for each coordinate, *p* < 0.007): the right M1_ROI peak was in a more lateral, posterior, and upper position than the left M1_ROI peak. Note that neither MP, nor sex, nor TIV had any effect on the M1_ROI location coordinates in each hemisphere, or on the location of the between-hemisphere difference.

**Table 2:** **Location of M1 activation maxima**.

	*x*	*y*	*z*
Right M1	−39.7 ± 3.9	−19.9 ± 3.7	54.7 ± 4.0
Left M1	38.8 ± 3.7	−19.0 ± 3.3	53.6 ± 4.0

*All coordinates are presented as mean ± SD*.

#### BOLD variations in M1_ROI according to movement side and manual preference

MP by movement side interaction had no significant effect on BOLD variations observed in the M1_ROIs contralateral to the moving finger (Table 3, *p* = 0.10). The trend was caused by higher but not significantly different activation values during LFTT in RH vs. LH (*p* = 0.23); there was no such difference during RFTT.

**Table 3:** **Mean BOLD values in M1 during each task in each hemisphere**.

	Right FTT		Left FTT	
	Right-handers	Left-handers	Right-handers	Left-handers
Right M1	−0.68 ± 0.53	−0.55 ± 0.53	2.87 ± 1.04	2.78 ± 0.97
Left M1	2.44 ± 0.92	2.49 ± 0.89	−0.36 ± 0.63	−0.51 ± 0.55

*All data are presented as mean ± SD*.

There were significant effect of educational level on contralateral activation (*p* = 0.009), the larger the educational level, the lower the activation in both tasks. There was also an effect of age in interaction with the side of the moving hand (*p* = 0.005): with increasing age a trend towards a significant decrease in M1 activation was present only during LFTT (LFTT: *p* = 0.09, RFTT: *p* = 0.16).

**Figure 5:**
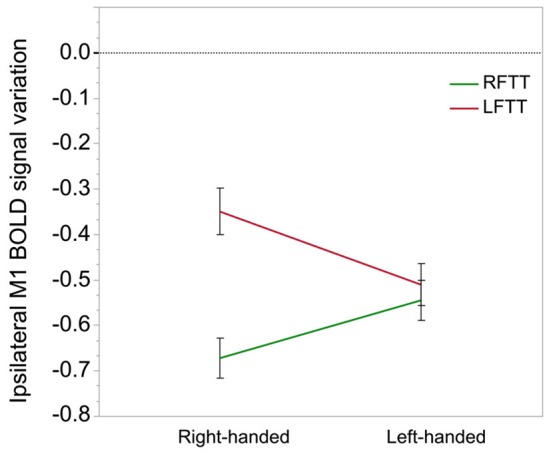
**Ipsilateral M1-ROI deactivation during finger tapping in right- and left-handers**. This graph illustrates the significant interaction observed between the side of finger tapping (right in green, left in red) and manual preference on deactivation in the ipsilateral M1_ROI (% of BOLD variation, arbitrary unit). The between-hand difference was significant in right-handers only (*p* = 0.0001) because they exhibit a highly significant difference in right and left hand movement ipsilateral deactivation because of a small deactivation during movement of the non-dominant hand, opposed to left-handers who do not display a comparable difference. Error bars represent one standard error from the mean.

In contrast, we observed that MP by movement side interaction had a significant effect (*p* = 0.0003) on BOLD variations in the M1_ROI ipsilateral to movement. From a movement side perspective, *post hoc* analysis showed that this effect was caused by greater deactivation in LH than in RH during LFTT (LH–RH mean difference = −0.15, *p* = 0.01; Figure 5) and greater deactivation in RH than in LH during RFTT (RH–LH mean difference = −0,14, *p* = 0.05). From an MP perspective (Figure 5), this interaction can also be described as the existence of a significant difference between ipsilateral deactivation during dominant and non-dominant hand movement in RH (mean = 0.32, *p* < 0.0001) but not in LH (mean = 0.03, *p* = 0.51).

Note that there were significant effects of sex and of educational level, males having higher deactivation (men − women = −0.21, *p* = 0.0002), and the higher the educational level the higher the deactivation (*p* = 0.006) on ipsilateral deactivation, without interaction with the side of hand movement.

#### Ipsilateral deactivations and asymmetry in manual ability

A significant interaction between movement side and asymmetry of manual ability was observed due to opposite profiles between RFTT and LFTT ipsilateral M1_ROI deactivation with increasing MSA index (interaction: *F* = 20.1, *p* = <0.0001).

We computed the same MANOVA, but this time entering MP and the interaction between MP and MSA index. MP main effect was significant (as previously described) but there was no interaction between MP and MSA index and the interaction between difference in deactivation with hand moving and MSA index remained significant (interaction *F* = 6.14, *p* = 0.01). There was neither interaction between movement side and manual ability when the M1_ROI contralateral to the movement side was considered (*p* = 0.53). Note that there was no effect of sex, age, educational level on this interaction.

**Figure 6:**
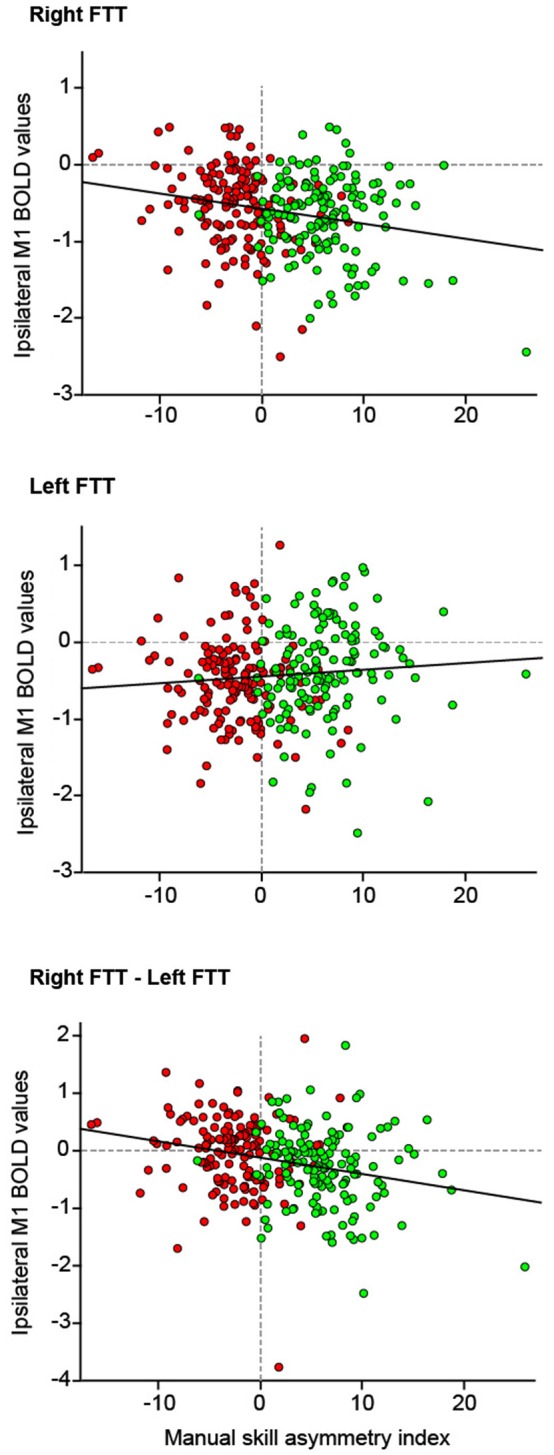
**The relationship between deactivation of the M1_ROI ipsilateral to the side of movement and the manual skill asymmetry index (*n* = 284 participants)**. Because there was no interaction between handedness and manual skill asymmetry index on the strength of deactivation the data of right- and left-handers are pooled (right-handers: green dots, left-handers: red dots). Top: right finger movement; Middle: left finger movement; Bottom: difference between ipsilateral M1-ROI deactivation measured during right finger movement and ipsilateral deactivation during left finger movements. Linear regression lines are in red.

*Post hoc* analysis pooling right- and left-handed showed that right M1_ROI deactivation amplitude increased significantly during RFTT as rightward asymmetry of manual ability increased (*R*^2^ = 0.05, *p* = 0.0001). During LFTT, the left M1_ROI deactivation amplitude decreased as rightward asymmetry of manual ability increased, although the regression was not significant (*R*^2^ = 0.01, *p* = 0.12). These opposite behaviors led to a significant correlation between MSA index and the difference in ipsilateral deactivation between RFTT and LFTT (*R*^2^ = 0.066, *p* < 0.0001, Figure 6).

### Summary of the results

The present results show that manual lateralization among LH and RH of the study population was characterized by larger differences in MSA in RH owing to poor performance with their non-dominant hand compared with LH, who exhibited a lesser difference in ability between their dominant and non-dominant hands. These results demonstrate that the activation intensity of the M1 hand area contralateral to the movement side is independent of which hand is moved and of MP. They also indicate that RH exhibit significantly greater deactivation of the M1 ipsilateral to the movement of their dominant hand than LH, and vice versa. Importantly, only RH exhibited significantly greater ipsilateral deactivation during their dominant hand movement compared with their non-dominant hand movement. Independently of handedness, the intensity of the ipsilateral deactivation during the right-hand movement correlated negatively with the asymmetry of manual ability; a trend towards the reverse pattern was present during LFTT, leading to significant positive correlation between asymmetry of manual ability and right-hand vs. left-hand movement difference in ipsilateral deactivations.

## Discussion

In the present study, the investigation of a large population balanced for handedness allowed investigators to uncover new elements regarding the neural support of hand motor dominance. The activity of participants’ motor cortices during unimanual finger movements revealed the importance of ipsilateral deactivation as a marker of MP. We will first discuss methodological issues. Then, we will comment on the absence of difference in contralateral M1 activity with respect to MP, as well as on ipsilateral M1 behavior in RH and LH.

### Methodological issues

In addition to its enhanced statistical power as a large study, a major strength of the present study comes from the absence of between-group and between-hand differences in the FTT frequency measured during the fMRI acquisition. The manual lateralization profiles of the investigated groups are consistent with previous studies on manual lateralization. Like others, we observed that LH are less lateralized in terms of manual ability than RH and demonstrated that this was because of their preserved right-hand performance, in contrast to the poor left-hand abilities of RH. We also noted that women, whether right or left-handed, were slightly more lateralized than men, a phenomenon previously reported by others (Tapley and Bryden, 1985). Another strength of our study is the use of an individual localizer for each hemisphere’s hand motor area, thus bypassing between-participant anatomical variability. It is interesting to note the slight but significant difference in inter-hemispheric M1 location that corresponds to the global torsion of the brain, the torque, which corresponds to the protrusion of the right frontal *petalia*. Since we did not use a symmetrical template, the imprint of the torque is still present although limited to a residual of 1 mm in each direction; however, M1 hand area location did not differ between RH and LH, suggesting little difference in M1 position with respect to handedness.

The limitation of this work is that we restricted ourselves to variations in M1 activity, while DCM has shown that the strong influence of SMA on M1 varies with handedness (Pool et al., 2014). However, the present fMRI paradigm was not designed for DCM analysis because there were only 6 blocks of FTT, three for the right and three for the left hand movement, the low repetition of the block preventing a causal analysis of the BOLD signal. The focus of the present work was rather to evaluate, for the first time, the importance of ipsilateral deactivations, which have been documented in RH by Hayashi et al. (2008) while not in LH. This study takes advantage of the large population of LH in the BIL&GIN database to show how ipsilateral deactivations of M1 are related to MP.

### Absence of variation of activation in M1 contralateral to the moving hand with manual preference

The present results demonstrate that the amplitude of M1_ROI activation in the M1 contralateral to the movement side is independent of the movement side and MP. Whole-brain analysis revealed very few differences in activity between LH and RH during either RFTT or LFTT. In addition, these differences were not located in the hand motor representation. One possible explanation for the lack of difference with handedness could be a ceiling effect of BOLD signal. As Hayashi et al. (2008) showed, contralateral M1s show a non-linear increase of BOLD signal according to the increase of movement frequency. When the movement frequency is relatively high, BOLD signal might not be sensitive enough to capture the differences in M1 activity. However in the present study the participants mean frequency was around 2.2 Hz, not yet corresponding to activation ceiling, while, on the opposite ipsilateral deactivation reach their ceiling at this frequency (Hayashi et al., 2008). This explanation is thus not sufficient to explain the absence of difference in activation with handedness while a difference in deactivation was detected despite of possible ceiling effect.

We noticed only two small clusters that were more activated during LFTT in the left hemisphere of RH compared with LH. One of these areas was the left FEF, a region belonging to the dorsal stream of the attentional network (Petit et al., 2009). One may first consider that, independent of the MP, subjects activated this attentional network due to a higher attentional engagement during the 12 s finger-tapping sequences while they had to tap and maintain the tapping at a given and internally guided rhythm, than during the reference condition during which they had simply to fixate the central cross. The present larger left FEF activation in RH is therefore related to the differences of lateralization of the dorsal attentional network between RH and LH. We recently demonstrated that RH show larger left activation of the dorsal attentional network than LH (Petit et al., 2014).

Considering the statistical power provided by the large sample of the present study, the lack of a significant difference between tasks and between handedness is consistent with previous reports that handedness has no effect on BOLD activity of M1 contralateral to movement (Kim et al., 1993; Civardi et al., 2000; Solodkin et al., 2001; Klöppel et al., 2007), even when movement frequency-dependent changes (Pool et al., 2014). Conclusively, hand preference does not find an expression in the activity of the M1 contralateral to hand movement during various unilateral hand movements.

### Ipsilateral deactivation of M1_ROI is greater in the group moving its dominant hand

Like Hayashi et al. (2008), we observed greater deactivation in homotopic ipsilateral M1 during dominant hand movement in RH. Hayashi suggested that the ipsilateral deactivation in the non-dominant M1 hand area during a dominant hand movement would be the physiological expression of the decrease in neural excitability caused by inhibitory afference through the corpus callosum. In addition to observations coming from paired-pulse TMS studies upper mentioned, such proposal is in accordance with observations of split-brain patients suffering from uncontrolled non-dominant hand behavior or having strange hand feeling during the acute phase after callosotomy (Zaidel, 1994), and with the fact that such patients are no longer able to learn complex bimanual movements during the chronic phase (Zaidel and Sperry, 1977). In the same vein, patients with congenital corpus callosum agenesis are reportedly incapable of motor differentiation between hands, which suggests that an inhibitory action through the corpus callosum is essential during ontogeny in order to acquire such differentiation (Dennis, 1976). Also consistent with Hayashi’s hypothesis is the recent report of very strong positive correlation between the strength of IHI measured by the ipsilateral silent period and the microstructure of callosal tracts connecting M1 (*r* = 0.76, Fling et al., 2013).

The present work also provides new insights regarding the relationship between ipsilateral deactivation and MP. The first is the observation of between-group differences in ipsilateral deactivation during dominant hand movement, with larger amplitude of ipsilateral reduction of M1_ROI activity in the group that is moving its dominant hand. However, the second is recognition of a handedness effect on the between-hands differences in ipsilateral M1 deactivations: in LH, the decrease of activity in the ipsilateral cortex was comparable for the movement of preferred and non-dominant hands, while in RH it was larger for the movement of the preferred hand.

### Left-handers have a strong motor specialization of both hands

The fact that an ipsilateral deactivation was not significantly reduced when LH moved their right hand compared with their left hand must be confronted with the fact that LH exhibit less difference in manual ability between hands than RH. Differences in manual ability between RH and LH are mainly due to the low left hand performance of RH, which might be related to smaller ipsilateral deactivation during their left hand movement than their right hand movement. In other terms, RH who do not exhibit ipsilateral deactivation when moving their left hand exhibit low performance with this hand. This is untrue for LH, who have better non-dominant hand skill than RH and exhibit comparable ipsilateral deactivation during movement of the either non-dominant or the dominant hand. One might say that while RH have a strong specialization of the left M1 during right hand movement, LH exhibit contralateral motor specialization during the movement of either hand.

These observations are in agreement with two recent paired-pulse TMS studies conducted by the same group. The first study used TMS during a bimanual task to demonstrate a larger IHI in RH than in LH after stimulation of the dominant cortex. The study also showed that LH motor cortices exhibit equal capacities, corresponding to comparable inhibitory processing, after TMS stimulation of the right or left M1 (Reid and Serrien, 2012). In their second investigation of ipsilateral M1 excitability (thus during unimanual hand movement), the same authors again showed greater inhibition from the left-dominant hemisphere to the right among RH than among LH, and that LH exhibited comparable behavior regardless of which hemisphere was stimulated (Reid and Serrien, 2014). As a whole, these results suggest that LH have two dominant motor cortices, which leads to lower asymmetry than RH, leading to a decreased manual lateralization.

### Between-hand difference in ipsilateral deactivation is associated with asymmetry in manual skill

In the present work, we also showed that the between-hand difference in ipsilateral deactivation varied linearly and negatively with the asymmetry in manual skill of the participants in the same way in RH and LH. The largest was the ipsilateral deactivation during a given hand movement, the larger was the MSA favoring the same hand. Actually, the negative linear relationship between the difference in deactivation between RFTT and LFTT and the MSA index corresponded to a negative difference in deactivation in RH with strong right hand manual asymmetry and a positive difference in LH with strong leftward asymmetry in manual ability. Considering that ipsilateral deactivation indices IHI, such correlation supports Hayashi’s hypothesis that a difference in strength of IHI is associated with manual lateralization. This finding also shows that ipsilateral deactivation, indexing IHI, is a mechanism common to RH and LH, with greater ipsilateral deactivation marking the most skilled hand, and corresponding to an increase in IHI with increasing cortical specialization. The strength of this correlation was moderate (it explained 6% of the variance) but one should keep in mind that other cortical areas interact with M1 and other factors are associated with variations in hand skill such as training, aging or expertize.

The advantage of the finger tapping test, as opposed for example to the peg-board, is that it is purely motor, the given instruction being to tap as quickly as possible. However, given that asymmetries in manual ability and MP strength were both evaluated, we also investigated the association of between-hand difference in ipsilateral deactivation with ES. Although ES is more variable in LH than in RH, the correlation between the ES and between-hand difference in ipsilateral deactivation was also significant (Spearman *r* = 0.27, *p* < 0.0001), thereby demonstrating its consistency across different measures of manual lateralization.

Finally, the well known increased variability in MP strength (Mellet et al., 2014) and lower asymmetry in manual ability (Peters and Durding, 1978; Doyen et al., 2001) of LH, also observed in the present sample, explains the fact that, as a group, LH did not exhibit greater ipsilateral deactivation when moving their dominant left hand. Such lower lateralization of LH resulted in a main effect of MP after controlling for MSA. However, similarly to RH, LH asymmetry in manual ability was related to the difference in between-hand lateralization and possibly of strength of IHI.

To conclude, the present study shows that the difference in ipsilateral deactivation explains part of the variance in MSA in both RH and LH, thereby validating the hypothesis proposed by Hayashi et al. (2008) that ipsilateral deactivation is a marker of hand lateralization. In addition, consistent with previous paired-pulse TMS studies, the present results also showed that LH are characterized by a cortical specialization for the movement of either hand, leading to less manual asymmetry than RH, who show stronger deactivation when their dominant hand is moving. These results underline the importance of ipsilateral deactivation in the setting up of motor skill asymmetry and call for investigations of their role during motor learning.

## Conflict of interest statement

The authors declare that the research was conducted in the absence of any commercial or financial relationships that could be construed as a potential conflict of interest.

## Footnotes

^1^www.gin.cnrs.fr

^2^www.fil.ion.ucl.ac.uk/spm/software/spm5

^3^www.jmp.com, SAS Institute Inc., 2012
